# The Case for Assessing the Drivers of Measles Vaccine Uptake

**DOI:** 10.3390/vaccines12060692

**Published:** 2024-06-20

**Authors:** Jessica Kaufman, Ashleigh Rak, Sophia Vasiliadis, Navrit Brar, Eeman Atif, Jennifer White, Margie Danchin, David N. Durrheim

**Affiliations:** 1 Vaccine Uptake Group, Murdoch Children’s Research Institute, Melbourne, VIC 3052, Australia; ashleigh.rak@mcri.edu.au (A.R.); sophia.vasiliadis@mcri.edu.au (S.V.); margie.danchin@rch.org.au (M.D.); 2 Department of Paediatrics, The University of Melbourne, Melbourne, VIC 3052, Australia; 3 Melbourne Medical School, The University of Melbourne, Melbourne, CIV 3052, Australia; nbrar@student.unimelb.edu.au (N.B.); eatif@student.unimelb.edu.au (E.A.); 4 Health Protection, Hunter New England Local Health District, Booth Building, Wallsend Health Services Longworth Avenue, Newcastle, NSW 2287, Australia; jennifer.white3@health.nsw.gov.au (J.W.); david.durrheim@health.nsw.gov.au (D.N.D.); 5 School of Medicine and Public Health, University of Newcastle, Newcastle, NSW 2308, Australia; 6 Department of General Medicine, The Royal Children’s Hospital, Melbourne, VIC 3052, Australia

**Keywords:** vaccination, vaccine uptake, measles, MMR, social science, barriers, vaccine hesitancy

## Abstract

Global measles cases are on the rise following disruptions to routine immunisation programs during the COVID-19 pandemic, with devastating consequences. According to the World Health Organization, the behavioural and social drivers of vaccination include what people think and feel about vaccines, social processes, motivation to vaccinate and practical barriers to vaccination. However, the drivers of measles vaccine uptake are not necessarily the same as those for other childhood vaccines, and we lack data on how these drivers specifically have changed during and since the COVID-19 pandemic. Without accurately measuring the behavioural and social drivers for measles vaccination, and ideally measuring them serially over time, countries cannot design, target and implement interventions that effectively increase and sustain measles vaccine coverage. This paper outlines what is and is not known about the behavioural and social drivers of measles vaccination and provides recommendations for improving their post-pandemic assessment.

## 1. Background

The current worldwide resurgence in measles cases is a critical global health issue. From 2021 to 2022, the estimated number of measles cases worldwide increased by 18% to 9,232,300, and the number of World Health Organization (WHO) countries experiencing large or disruptive outbreaks increased from 22 to 37 out of 194 [1]. In the same period, estimated measles deaths increased by 43% to 136,200, and the first months of 2024 demonstrate the perpetuation of these disturbing trends [1].

While measles is among the most infectious communicable diseases in the world, it is also preventable [2]. The measles vaccine has saved more lives than any other vaccine [1,3]. However, very high coverage—over 95% of the population—is required to prevent devastating outbreaks [4]. Since the start of the COVID-19 pandemic in 2020, routine childhood vaccination rates have dropped 3% worldwide [5]. The detrimental impact was particularly pronounced for measles: global first dose measles-containing vaccine (MCV) coverage declined from 86% pre-pandemic to 81% in 2021, the lowest coverage since 2008 [1]. Despite some early recovery in 2022, 40 million children missed a measles vaccine dose [1]. The greatest declines have been seen in low- and middle-income countries [6], but high-income countries have also been impacted [7,8,9].

The reasons for this decline are multifactorial. Deployment of immunisers away from childhood vaccination to delivering COVID-19 vaccines to older age groups had an immediate impact. However, while childhood immunisation services have now been restored, coverage has not bounced back to pre-pandemic levels. Vaccine confidence and trust have also been impacted by the pandemic; in 2021, confidence in vaccines declined in almost all countries, according to data from the Vaccine Confidence Project [5]. As the World Health Organization Regional Verification Commission Chairs warn, the global drop in routine childhood vaccination threatens progress towards measles elimination and puts millions of children at risk of measles and other life-threatening vaccine-preventable diseases like polio and pertussis [6,10].

## 2. The Behavioural and Social Drivers of Measles Vaccination

Measles is one of many serious vaccine-preventable diseases, but the factors affecting measles vaccine uptake are not necessarily the same as those for other childhood vaccines. Coverage data can only tell us which locations or demographic groups have low uptake, but not why. It is now imperative that we understand the social and behavioural drivers of measles vaccination—separate from other childhood vaccines—so countries can develop tailored, cost-effective solutions for increasing coverage. 

A number of frameworks have been proposed to explain the drivers of vaccine uptake. For example, the 3Cs (highlighting Convenience, Complacency and Confidence) is one of the foundational models of vaccine hesitancy [11], while the 5As (Access, Affordability, Awareness, Acceptance and Activation) was designed specifically for application by diverse stakeholders to plan and target interventions [12]. The WHO Behavioural and Social Drivers (BeSD) of Vaccination framework includes many of the same social and behavioural drivers as these other models, organised into four categories: what people think and feel about vaccines, social processes, motivation to vaccinate and practical barriers to vaccination [13,14]. Uniquely, however, the BeSD framework is accompanied by both qualitative and quantitative data collection instruments and comprehensive supporting guidance for measuring these drivers. Without accurate measurement and, ideally, measuring serially over time, it is easy to assume that the main barriers are hesitancy or misinformation, when the actual issue may be access or availability or a complex mix of multiple factors. Below, we outline what is and is not known about the behavioural and social drivers of measles vaccination.

### 2.1. Thinking and Feeling

The way people think and feel about measles vaccination continues to be influenced by the discredited 1998 Lancet study reporting fraudulent data linking the measles, mumps and rubella (MMR) vaccine to autism. Although this study was formally retracted by the Lancet more than a decade ago, its pervasive impact remains. For example, studies from Australia and Saudi Arabia have found that up to 10% of parents are still concerned about a potential link between autism and the MMR vaccine [15,16]. A review of studies from the United States found that fear of autism was the most frequently cited reason for hesitancy about the MMR vaccine [17]. This was also the primary concern cited by over 70% of surveyed Somali parents in Minnesota, where a significant measles outbreak occurred in 2019 [18]. While a recent Australian study found that the belief that vaccines cause autism increased from 8.7% in 2017 to 14.4% in 2023 [19], very few other studies have compared evidence of pre- and post-pandemic beliefs, highlighting the need for repeated assessment of drivers of under- and non-vaccination.

Vaccine safety concerns are a significant factor in parents’ decision-making about measles vaccines. Some parents seek alternative vaccination schedules that delay measles vaccination or separate the components of the MMR vaccine [20], despite the risks and lack of supportive evidence for this practice [21]. Measles coverage can also be affected by vaccine safety signals about other vaccines that impact confidence in all childhood vaccines [22]. In the Philippines, the Dengvaxia vaccine for dengue fever caused the death of 14 vaccinated children out of 800,000 vaccinated in 2016–2017, after which the vaccine campaign was stopped [23]. Vaccine confidence plummeted: while 93% strongly agreed that vaccines were important in 2015, this figure dropped to just 32% in 2018 [24], and MCV1 coverage declined to 77% [25]. The resulting measles outbreak resulted in over 47,000 cases with more than 630 measles deaths in 2019 [26]. While coverage of DTP1 had recovered to 75% in the Philippines by 2022, MCV1 coverage remained worryingly low at 69% [25]. 

### 2.2. Social Processes

Measles vaccine decision-making is influenced by external social processes. For example, the origins of the modern anti-vaccine movement can be largely traced to the media coverage of the MMR controversy of the 1990s [27,28]. The MMR vaccine itself has been singled out by anti-vaccine activists and influencers [29], whose goal is to shape social norms. Religious leaders have also exerted significant influence on measles vaccination. In 2018, a fatwa was issued by Islamic clerics in Indonesia stating that the measles-containing vaccine was haram, or made using forbidden processes or ingredients. This caused vaccination rates to drop significantly across the country and especially in Aceh, a province ruled by sharia law [30]. Similarly, a study from Thailand found that religious opposition was the primary barrier to measles vaccination [31].

One of the most significant measles outbreaks of recent years, in Samoa, was caused by a policy decision. Following two fatal vaccine reconstitution errors, where a muscle relaxant was accidentally used, the country temporarily suspended the entire measles vaccination program. This decision dramatically undermined confidence in vaccination, and the country’s ongoing measles outbreak worsened, with 83 deaths in children due to a drop in measles coverage [32]. Understanding the specific social processes that affect measles vaccination decisions is critical, as these may differ between vaccines.

### 2.3. Practical Issues

The practical issues affecting vaccine uptake are multifactorial and can be unique in relation to measles vaccination. For example, stock-outs of measles vaccines specifically have been a major challenge in countries like Romania [33]. The vaccine is heat-sensitive, requiring meticulous cold chain management and reconstitution that complicates storage and delivery and increases the risk of secondary vaccine failure [34]. As a live attenuated vaccine, it can be more reactogenic than inactivated vaccines. The side effects are usually delayed 5 to 10 days, with fever and a measles-like rash occurring in 10–25% of recipients [35]. Even though these side effects are generally mild, parents frequently cite the challenge of taking time off work to care for a child after vaccination as a barrier to uptake [36]. 

## 3. What Do We Know about How the Pandemic Has Changed the Drivers of Measles Uptake?

For the reasons outlined above, it is conceivable that measles vaccination has been differentially impacted by the pandemic in many unique and important ways. However, despite the urgent global concern about measles resurgence and inadequate measles vaccine coverage, there is a gap in our understanding of how the social and behavioural drivers of measles vaccination have changed during and since the COVID-19 pandemic. While many studies assessing factors that influence measles vaccine uptake have been published since 2020, most report pre-pandemic data [37,38,39]. Of the few that did report data collected since the start of the pandemic, lack of knowledge about MMR was a commonly cited issue [31,40]. These studies highlight the need for ongoing parental education on the measles vaccine and the protection it can provide to children and their communities. However, educational interventions alone may not be sufficient to improve uptake if there are more complex barriers, such as low levels of vaccine literacy [41], or if other barriers like access issues remain in place [42,43]. This is why it is critical to repeatedly and systematically assess a range of systemic factors and social and behavioural drivers over time to identify growing problems and lingering barriers.

We need serial data on drivers of measles vaccination—and granular data on MCV uptake—if we are to design, target and implement interventions that effectively increase and sustain measles vaccine coverage. It may be that these drivers are the same as for other childhood vaccines, but, given the importance and threat measles poses, we cannot assume this to be the case. We must make concerted efforts to understand the drop-off in measles coverage in different demographic groups and settings. 

Validated tools focused on social or behavioural factors associated with measles vaccine uptake are very limited. Some studies have adapted the Parent Attitudes towards Childhood Vaccination (PACV) scale [37] or the WHO Vaccine Hesitancy Scale (VHS) [44], and one MMR attitudinal scale has been validated in the UK [45]. However, all of these instruments focus on acceptance issues alone and do not capture data on practical access issues. The WHO Tailoring Immunization Programmes approach employs the COM-B behaviour change wheel to qualitatively assess barriers to uptake, including practical issues [46], but this approach is complex, resource intensive, and can be difficult to deploy rapidly and repeatedly. The WHO BeSD tools address both access and acceptance issues and have been designed for use globally, particularly in low- and middle-income countries, but they are not customised for individual vaccines. 

Using validated tools to understand and address the drivers of vaccination is a global priority [13]. There is now an urgent need to adapt these or other instruments to capture measles-specific data on a routine basis or when there are precipitous drops in coverage. While academic researchers may be best suited to conduct full psychometric validation of new or adapted tools, governments and public health organisations should at least look to existing validated tools rather than creating them from scratch. Ideally, existing tools should be subject to both forward and back translation to the required language and pilot tested with the target population to ensure clarity and relevance [47]. Serial surveillance of social and behavioural drivers of vaccination should be conducted alongside vaccine uptake monitoring. Coverage data from national immunisation registers, integrated with data from multiple agencies, show gaps in specific demographic and socioeconomic groups and locations but provide no data on why the gaps exist. The Immunization Agenda 2030 includes the concrete indicator of achieving 90% global coverage for MCV2 and achieving measles elimination in every WHO region [48]. The WHO Joint External Evaluation tool also has a first dose MCV indicator, but we strongly recommend countries consider indicators for vaccine drivers, with a focus on measles.

With these data, countries can apply qualitative approaches to tailor interventions to address country and sub-national specific measles concerns.

We make the following recommendations:

- At a macro level, health departments should invest in and encourage a learning health systems approach to systematically collect data and apply it to inform evidence-based care and decision-making [49,50,51]. Some academic institutions offer online short courses and fellowship programs for low- and middle-income country leaders [52,53].
- Countries should ideally establish serial surveillance of the social and behavioural drivers of measles vaccine uptake (e.g., vaccine confidence, concerns and social influences) over time. Short-form instruments, such as the five-item BeSD key indicators, may be suitable for embedding in existing population surveys. Partnerships with academic institutions should be considered for local validation of measurement instruments.
- Interventions should be evidence-based and targeted to address specific barriers identified through surveillance. A recent systematic review and meta-analysis maps behavioural interventions against the BeSD domains, identifying provider recommendations (social processes domain) and on-site vaccination (practical issues domain) as the most effective strategies to improve uptake [54].
- In addition to aligning with identified barriers, interventions should be locally tailored through community co-design. Measles vaccine uptake may be particularly susceptible to concerns about vaccine safety. Community-led and -owned approaches to encourage and sustain vaccine demand are critical, such as building the capacity of health workers, community leaders and faith leaders to advocate for vaccination [54,55]. These groups are often highly trusted and influential but benefit from specific vaccine education and vaccine communication skills training.
- Assessing perceived vaccine accessibility, affordability and availability is critical, even in settings where vaccinations are provided free of charge [56]. Governments should prioritise guaranteeing availability and optimal supply of vaccines. Officials should employ evidence-based risk communication and response approaches to address vaccine safety signals while maintaining public trust.
